# Occurrence and concentration variability of pharmaceuticals, pesticides, and polyphenols in Nyabugogo River, Rwanda

**DOI:** 10.1007/s10661-026-16028-z

**Published:** 2026-10-15

**Authors:** Brigitte Mukarunyana, Elias Nyandwi, Telesphore Kabera, Christoffer Boman, Jerker Fick

**Affiliations:** 1 Department of Chemistry, Faculty of Science and Technology, Umeå University, SE-901 87 Umeå, Sweden; 2 College of Science and Technology, University of Rwanda, Po Box 3900, Kigali, Rwanda; 3 Department of Applied Physics and Electronics, Umeå University, SE-901 87 Umeå, Sweden

**Keywords:** Organic contaminants, Nyabugogo River contamination, Urban discharges and agricultural run-off, Spatiotemporal variability, Rwanda

## Abstract

Pharmaceuticals, pesticides, and polyphenols are increasingly detected in aquatic environments, impacting water quality and aquatic life. This study screened these contaminants in Rwanda’s Nyabugogo River, analysing 45 water samples from rural, peri-urban, and urban areas using solid-phase extraction and LC–MS/MS. Fifty-seven pharmaceuticals, five pesticides, and four polyphenols were quantified, with the highest average concentrations of albendazole (960 ng/L), metalaxyl (70 ng/L), and caffeic acid (49 ng/L). Pharmaceutical levels were significantly higher in urban areas, reflecting inadequate sanitation and wastewater management in Kigali, while pesticide and polyphenol concentrations varied across different types of land use, influenced by agricultural and urban discharges. Temporal sampling revealed significant within-day concentration fluctuations but consistent day-to-day levels, indicating persistent contamination sources. These findings provide essential baseline data for monitoring organic pollutants and informing strategies to mitigate contamination in urban river systems.

**Supplementary Information:**

The online version contains supplementary material available at https://doi.org/10.1007/s10661-026-16028-z.

## Highlights

- First assessment of organic contaminants in Nyabugogo River
- Pharmaceutical levels show rural-urban gradient
- Urban and agricultural inputs shape pesticide and polyphenol distributions
- Daily concentration variability driven by sanitation practices

**Graphical Abstract:**

**Supplementary Information:**

The online version contains supplementary material available at https://doi.org/10.1007/s10661-026-16028-z.

## Graphical abstract

**Figure:**
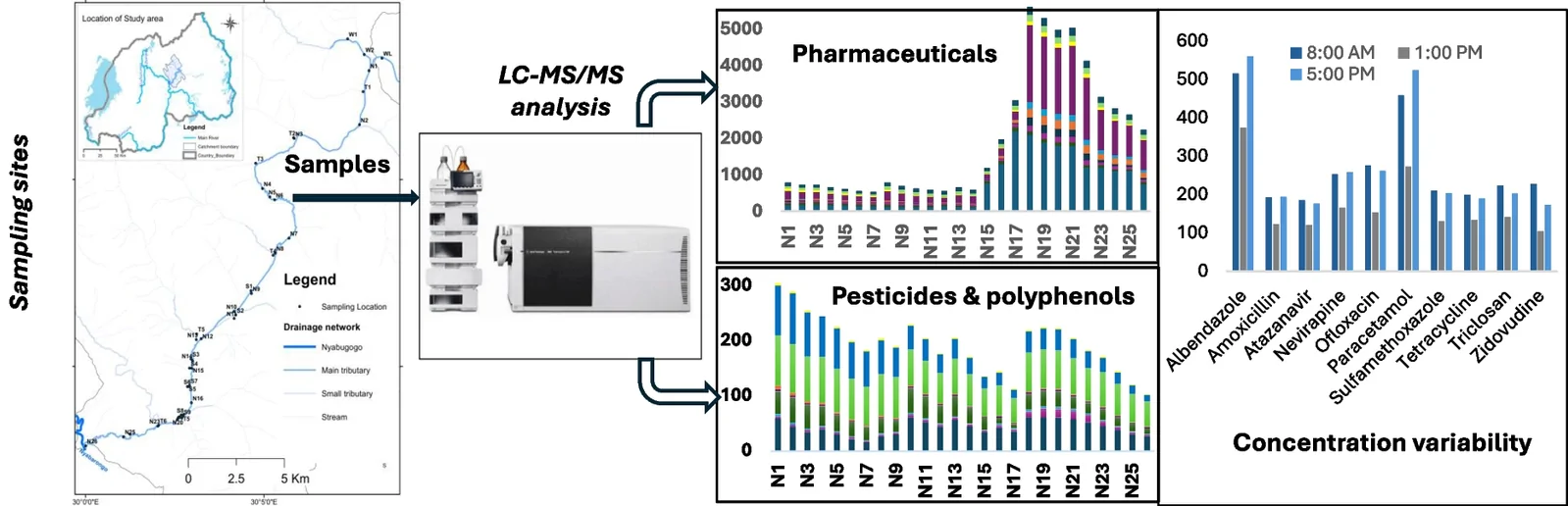

## Introduction

The large-scale commercial production and use of human-made chemicals have led to the widespread release of harmful organic substances into river systems (Mishra et al., 2023; Wilkinson et al., 2022). Pharmaceuticals, pesticides, and polyphenols are among the most frequently detected contaminants in surface water, groundwater, and even drinking water, primarily due to their extensive use and inadequate disposal practices (Hejna et al., 2022; Mishra et al., 2023; Schwanen et al., 2023).

Pharmaceuticals enter the environment primarily through human and animal excretion, hospital and industrial discharges, and improper disposal of unused or expired medications (Anand et al., 2022; Larsson, 2014). A large proportion of pharmaceuticals consumed are eliminated either unchanged or as metabolites, which may retain similar or different characteristics compared to the original substances (Justyna & Zimmermann, 2022), and subsequently enter aquatic ecosystems through direct wastewater discharges or via discharges from municipal wastewater treatment plants (Anand et al., 2022; Justyna & Zimmermann, 2022). However, even treated effluent has been shown to contain elevated levels of pharmaceuticals since most conventional treatment techniques have limited removal efficiency (Samal et al., 2022). Pesticides reach surface water mainly through agricultural runoff, spray drift, and leaching. Additionally, soil erosion in agricultural areas can transport pesticide-contaminated sediment into water bodies (Okoye et al., 2022; Zhou et al., 2025). Polyphenols originate from plants, but their presence in surface water can also result from anthropogenic activities, including agricultural runoff and discharges from industries such as pulp and paper, coffee processing, and olive washing stations (Lou et al., 2025; Wu et al., 2022). Additional sources include domestic wastewater and landfill leachate (Ndwabu et al., 2023). Concentrations of pharmaceuticals, pesticides, and polyphenols in surface water generally fall within the ng/L to μg/L range. These concentrations are associated with diffuse sources, including municipal wastewater effluents, agricultural runoff, and urban runoff (Mishra et al., 2023; Ndwabu et al., 2023; Wilkinson et al., 2022).

The ecological implications of pharmaceuticals, pesticides, and polyphenols have become an important focus of contemporary ecotoxicology. Pharmaceuticals, for example, can alter endocrine function, behaviour, metabolism, and reproduction in aquatic organisms, even at concentrations far below therapeutic doses (Anand et al., 2022; Larsson, 2014; Wilkinson et al., 2022). Pesticides, by design, are biologically active and can impair aquatic primary producers such as algae, phytoplankton, and aquatic plants (Abu Qamar et al., 2024). Insecticides can disrupt neurological function, leading to mortality or altered behaviour in invertebrates and fish, while herbicides can inhibit photosynthesis and reduce primary productivity, thereby influencing aquatic food webs. Fungicides may disrupt microbial communities that are essential for nutrient cycling (Abu Qamar et al., 2024; Gruss et al., 2025). Polyphenols can inhibit microbial growth, alter algal and plant physiology, and induce oxidative stress in aquatic organisms (Mumtaz et al., 2024; Ndwabu et al., 2023).

Several pharmaceuticals, pesticides, and polyphenols are persistent in aquatic ecosystems. Many pharmaceuticals are designed to resist metabolic and chemical degradation, thereby maintaining therapeutic activity in the human body (Gajula et al., 2021). These same properties chemical stability, low biodegradability, and resistance to hydrolysis and photolysis enable compounds such as antibiotics, antiretrovirals, and non-steroidal anti-inflammatories to persist in water bodies for prolonged periods (Anand et al., 2022; Gajula et al., 2021; Larsson, 2014). Likewise, numerous pesticides are formulated to resist environmental breakdown, providing long-lasting agricultural protection (Abu Qamar et al., 2024; Pathak et al., 2022). By contrast, polyphenols are generally more biodegradable; however, their persistence increases at high concentrations, when dissolved oxygen is limited, or when they bind to organic matter or sediments (Melo et al., 2005). Focusing on persistent compounds does not provide the full picture, as almost all of these are also pseudo-persistent, i.e., they are constantly present in certain aquatic environments due to constant release (Schwanen et al., 2023). Another complicating factor is the wide range of released pollutants, which can cause synergistic toxicity when combined, resulting in greater-than-expected harm and posing major risks to aquatic biodiversity (Mishra et al., 2023; Picó et al., 2020). This results from overlapping biological targets, cumulative stress, and complex interactions among pharmaceuticals, pesticides, polyphenols, and other organic pollutants (Gruss et al., 2025). Together, these interacting pollutants suppress primary producers, destabilize food-web relationships, reduce species richness, and impair ecosystem recovery capacity, making contaminant mixtures a major driver of biodiversity decline in aquatic environments (Alameddine et al., 2021; Mishra et al., 2023).

Although the presence of these contaminants has been increasingly documented in aquatic systems globally (Schwarzenbach et al., 2006; Sharma et al., 2019; Wilkinson et al., 2022), information from sub-Saharan Africa remains limited, especially in rapidly urbanizing catchments where wastewater treatment and environmental monitoring capacity may be constrained. (Munzhelele et al., 2024; Okoye et al., 2022). These concerns are heightened by the increased use of pharmaceuticals in recent decades, improved agricultural practices, and the growth of the industrial sector (Okoye et al., 2022).

In Rwanda, existing investigations of river water quality have predominantly focused on conventional physicochemical and microbiological parameters, such as chemical oxygen demand (COD), biological oxygen demand (BOD), nutrients (NH_4_^+^ and PO_3_^2−^), and selected inorganic contaminants, while little attention has been given to organic contaminants. To our knowledge, no studies have identified and quantified organic contaminants in Rwanda's rivers. Consequently, there is insufficient information on the identities, concentrations, and spatial distributions of these contaminants in the river systems. Generating such information is therefore essential for establishing baseline occurrence data, identifying contamination patterns and potential sources, strengthening environmental monitoring, and providing scientific evidence to inform pollution-management strategies in Rwanda.

This knowledge gap is particularly relevant to the Nyabugogo River, which drains a highly urbanized, intensively used catchment and receives inputs from domestic, agricultural, commercial, and other anthropogenic activities (Omara et al., 2020; Sekomo et al., 2014). The Nyabugogo River is an important tributary of the Nyabarongo River, joining it shortly before the confluence with the Akanyaru River to form the Akagera River, which drains 85% of Rwanda’s water into the Nile Basin via Lake Victoria (Julien et al., 2023; Omara et al., 2020). Previous studies indicate that the ecological health of the Nyabugogo River is declining rapidly due to dynamic land-use changes from rural to peri-urban and urban areas, the release of untreated sewage, plastic debris, industrial waste, and sediment accumulation from erosion (Omara et al., 2020; Sekomo et al., 2014). This study aims to analyse pharmaceuticals, pesticides, and polyphenols in the Nyabugogo River, its tributaries, and channels discharging into the river, using an analytical method that covers a broad spectrum of these compounds.

## Materials and methods

### Description of sampling area

The Nyabugogo River stretches 46 km (Nzabonantuma et al., 2015) from the outlet of Lake Muhazi to its confluence with the Nyabarongo River (from 1° 47′ 06″ S and 30° 07′ 57″ E to 1° 57′ 17″ S and 30° 00′ 24″ E). The Nyabugogo catchment area (1,661 km^2^) covers rural, peri-urban, and urban areas, representing 6.31% of Rwanda’s total surface area (26,338 km^2^) (Ministry of Environment, 2018). The number of people living within the catchment has been estimated at 1,400,000, with 47% in urban and peri-urban areas and 53% in rural areas (NISR, 2023). Upstream rural areas in Rwanda’s Northern and Eastern Provinces have population densities of 340–630 inhabitants per km^2^ and are predominantly agricultural, with maize, bean, vegetable, sugarcane, and rice cultivation, complemented by small-scale quarrying and cassiterite mining, as well as infrastructure including local markets and health centres (NISR, 2023; Republic of Rwanda, 2020). Peri-urban areas (1100–1700 inhabitants per km^2^) feature mixed land use, combining agriculture (rice, maize, and vegetables), small-scale quarrying, and limited industrial activities, including the Kabuye sugarcane factory, as well as markets and health facilities (NISR, 2023). Urban areas in Kigali have high population densities (7600–17000 inhabitants per km^2^) and are characterized by concentrated industrial activities, extensive social infrastructure, and localized urban agriculture in the Nyabugogo wetland near the Nyabarongo River confluence (NISR, 2023).

### Sampling sites and sampling

**Fig. 1:**
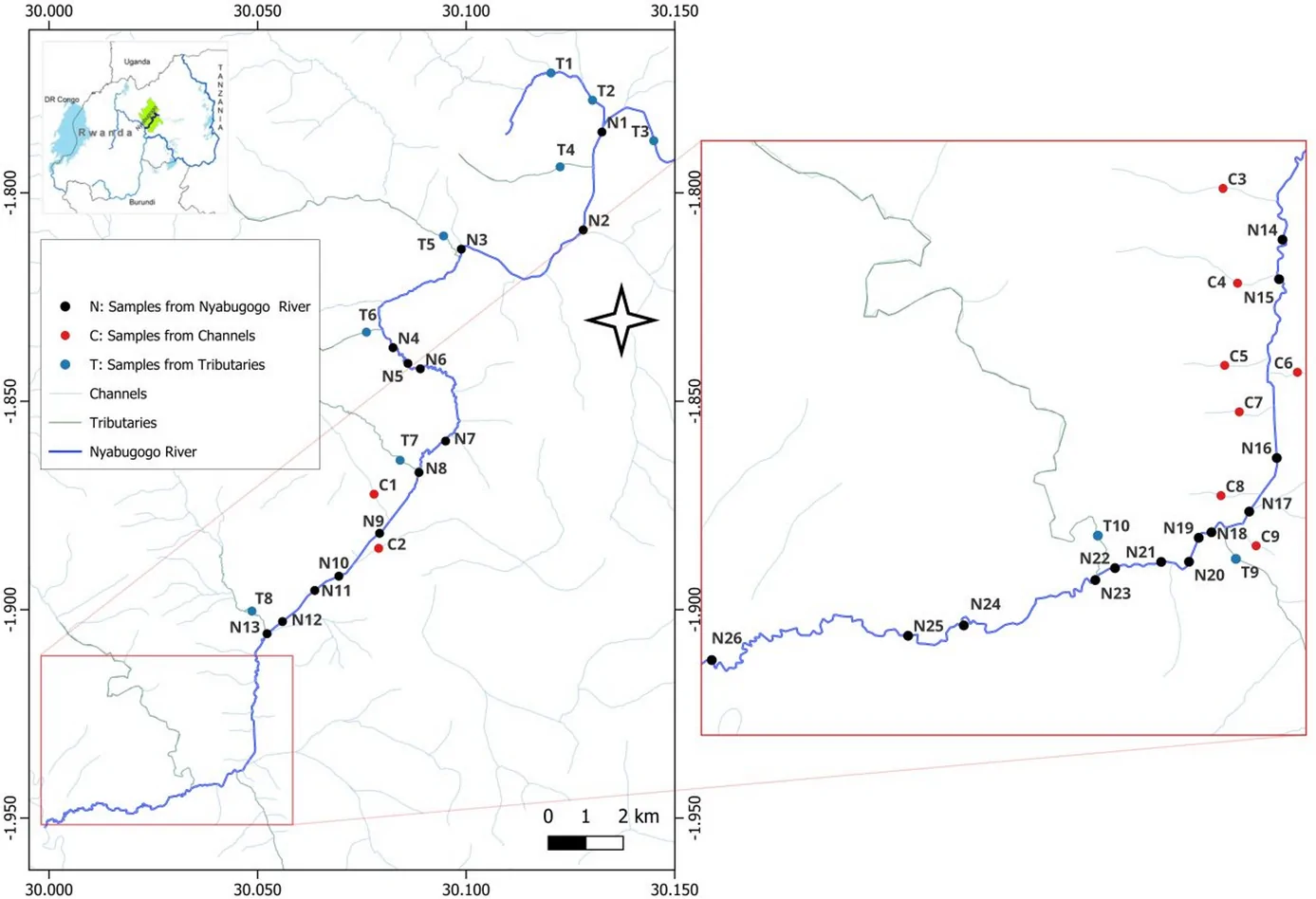
Sampling points, water from tributaries (**T**), water from channels and small drains (**C**), and water from the Nyabugogo River (**N**). In total, 45 water samples were collected: 26 water samples from the river (N1-N26), 10 water samples from tributaries (T1-T10), and 9 water samples from channels (C1-C9)

Sampling sites were selected based on the delineation of the Nyabugogo River in GIS. A total of 45 water samples were collected: 26 from the Nyabugogo River, from N1 (upstream, at its origin) to N26 (downstream, where it meets the Nyabarongo River), ten from tributaries (T1 to T10), and nine from channels or drains (C1 to C9), Fig. 1. In tributaries and channels, water samples were taken ten metres before their confluences with the river, and corresponding river samples were taken approximately one hundred metres downstream.

Sampling sites N1–N6 and T1–T6 are in rural areas, N7–N14, T7, and C1–C2 are situated in the peri-urban area, and N15–N26, T8–T10, and C3–C9 are urban sites. From N14 to N26, the river traverses the Nyabugogo wetland. Sampling point locations and coordinates are provided in Fig. 1 and Table S1 in the Supplementary Information, respectively.

Sampling occurred over three consecutive weeks, from 6 to 24 January 2025, each day from 8 a.m. until 5 p.m. One 450 mL sample was collected at each site. Additionally, samples were collected from site N21, located in the urban area of the Nyabugogo River in the Nyabugogo Centre, to monitor variations in concentrations of targeted organic pollutants. Sampling was conducted at N21 over five consecutive days at 8 a.m., 1 p.m., and 5 p.m. Water samples were collected at depths of 15 to 60 cm to avoid the surface layer at each site.

The sampling process involved rinsing a 500 mL polyethylene bottle three times with water from the site, then submerging it and filling it with approximately 450 mL. At each water sample collection site, the geographical coordinates were recorded using Google Earth application. The samples were quickly placed in cool boxes at around 4 °C and transported within two hours to the chemistry laboratory at the University of Rwanda. They were stored at −18 °C for no more than two days until pre-treatment. The pre-treated samples were then stored at −18 °C for no more two weeks and analyzed at Umeå University’s environmental chemistry laboratory in Sweden.

### Chemicals

All chemical standards, including the 24 internal standards used in this study, were classified as analytical grade (95–99.8%). Stock solutions for each analyte were prepared using methanol or acetonitrile and stored at −18 °C. This study included 158 compounds: 145 pharmaceuticals, 9 pesticides, and 4 polyphenolic compounds, selected based on regional use. LC/MS grade methanol and acetonitrile (LiChrosolv hypergrade) were obtained from Merck (Darmstadt, Germany). To produce purified water, a Milli-Q Advantage system with an ultraviolet radiation source (Millipore, Billerica, USA) was used. Formic acid (Sigma-Aldrich, Steinheim, Germany) at 0.1% was used to prepare the mobile chromatographic phases.

### Solid-phase extraction and LC–MS/MS analysis

Pretreatment was performed using solid-phase extraction (SPE). Before carrying out the SPE, 5 ng of internal standards containing labelled compounds were added to each sample (450 mL). The Oasis HLB cartridges were conditioned using 5 mL of methanol and 5 mL of Milli-Q water. Samples were then loaded into the cartridges at a relatively low flow rate (0.1 mL/s) to allow analytes to bind to the stationary phase. Finally, analytes were eluted using 5 mL of methanol. The solution from the extraction of each sample was then evaporated to approximately 25 µL and reconstituted with methanol to 1 mL in LC–MS vials. Samples were analysed using ultra-high-performance liquid chromatography coupled with tandem mass spectrometry (UHPLC-MS/MS). A Dionex UltiMate 3000 UHPLC system, which comprises two liquid chromatography pumps (an Ultimate LPG 3400SD quaternary pump and an HPG 3400RS binary UHPLC pump), an analytical column (Thermo Scientific Hypersil GOLD Aq 100 × 2.1 mm, 3 μm), and a precolumn (Hypersil GOLD, 10 × 2.1 mm, 3 μm), all connected to a TSQ Quantiva triple quadrupole mass spectrometer (Thermo Scientific), was used to analyse samples. A heated-electrospray ionization (HESI) source was utilized in positive or negative ionization modes. Chromeleon Xpress (Thermo Scientific) was utilized to manage the UHPLC system. The chromatographic separation was conducted using a gradient of Milli-Q water and methanol (LiChrosolv, Merck, Darmstadt, Germany). The mobile phase featured a gradient containing 0.1% formic acid in Milli-Q water and 0.1% formic acid in methanol. The resolution for both quadrupole instruments was adjusted to 0.7 full width at half maximum height. The voltage for the spray was adjusted to 3500 V with sheath gas at 40 arbitrary units, sweep gas at zero arbitrary units, ion transfer tube temperature at 350 °C, and vaporizer temperature at 338 °C. Two MS/MS transitions were tracked for each analyte: one for quantification and another for qualification. Peak identification was performed by comparing sample components with those of internal standards using Xcalibur™ 4.3 software (Thermo Fisher Scientific). To evaluate linearity and carry out quantification, an eight-point calibration curve ranging from 10 to 1000 ng/L was utilized. The transitions, precursors, and collision energies of pesticides and polyphenols analysed in this study are presented in Table S2 of the supplementary materials.

#### Quality assurance, quality control, limit of quantification, and calculations of concentrations

To detect potential contamination and carry-over, procedural and instrumental blanks were injected at regular intervals throughout the analytical sequence. Carry-over was further evaluated by injecting standards at 500 ng/L, followed by three injections of mobile-phase blanks. Quantification was based on comparing two transitions for each analyte, ensuring that their transition ratio remained within ± 30% of the ratio observed in the corresponding calibration standard. The retention times of the analytes had to be within ± 2.5% of those observed in the calibration standards. The limit of quantification (LOQ) was set to the second-lowest concentration within the linear range of the calibration curve. To obtain the concentrations of targeted analytes, a fixed quantity of an internal standard (IS) was added to each calibration standard and to the unknown samples. The analyte peak area was divided by that of the internal standard to obtain an area ratio, thereby reducing errors due to sample preparation, extraction recovery, and instrument variability. A calibration curve was obtained by plotting the area ratios against the known analyte concentrations. The concentration of an unknown sample was found by substituting the measured area ratio into the calibration curve equation and solving for the concentration. When the sample was diluted during preparation, the calculated concentration was multiplied by the dilution factor (DF) to obtain the final concentration. To evaluate method accuracy and analytical performance, a laboratory control sample (LCS) was prepared using Milli-Q water spiked with 1000 ng/L of the target pharmaceuticals, pesticides, and polyphenols, along with 5 ng of internal standards. The analysis was repeated 6 times, and the relative standard deviation (RSD) and recoveries were calculated. Recovery was calculated as the percentage of the measured concentration relative to the spiked concentration. Recoveries within 70–120% and RSD ≤ 20% were considered acceptable. River water samples were analysed using the same extraction, preparation, and LC–MS/MS procedures. The LC–MS/MS parameters, quality assurance, and quality control (QA/QC) values are presented in Table S3 of the supplementary information.

### Statistical analysis

For the observed pharmaceuticals, pesticides, and polyphenols, the following characteristics were measured: average (Av) and standard deviation (SD). For site N21, where data were collected for five consecutive days and three times a day, morning (8 a.m.), noon (1 p.m.), and evening (5 p.m.), averages and SD were computed for each pharmaceutical, pesticide, and polyphenol at each time of collection. A comparative analysis, t-test, and ANOVA were conducted to test the significance of differences in the average concentrations of detected compounds between rural, peri-urban, and urban areas, and in the times and days of collection. For this analysis, a 5% level of significance was used.

## Results and discussion

### Analysis of water samples

The average concentrations (*n* = 45) ranged from < LOQ to 960 ng/L for pharmaceuticals, 1.9 to 70 ng/L for pesticides, and 3.6 to 49 ng/L for polyphenols. The maximum concentrations detected were 4200 ng/L for pharmaceuticals (albendazole) at point C9, 130 ng/L for pesticides (metalaxyl) at point C9, and 190 ng/L for polyphenols (ferulic acid) at point C2, as presented in Table S4 and Table S5 of the supplementary information. Frequencies are calculated based on values above LOQ, and ranged from 67 to 100% for water samples from the river (N1-N26) (see Table S2 and S3 in supplementary information).

Of the 145 targeted compounds, 57 pharmaceuticals were detected and quantified, representing five major groups and 21 therapeutic classes under the Anatomical Therapeutic Chemical (ATC) classification system. Detected compounds comprised 12 antibacterials, eight antiretrovirals, and four antimycobacterials; nine cardiovascular agents (including calcium channel blockers, angiotensin-related agents, and beta blockers); and eight nervous system drugs (psycholeptics, psychoanaleptics, and antiepileptics). Additional detections included five respiratory drugs (mainly antihistamines), four musculoskeletal anti-inflammatories, four alimentary tract and metabolism agents (stomatologicals, antacids, and antidiarrheals), two dermatological agents, and one antihelminthic. These results indicate a complex mixture of pharmaceuticals exerting diverse contamination pressures on the Nyabugogo River system. In the river samples (*n* = 26), average concentrations ranged from < LOQ ng/L to 740 ng/L. The five pharmaceuticals with the highest concentrations were albendazole, paracetamol, triclosan, tetracycline, and nevirapine, with average concentrations of 740 ng/L, 620 ng/L, 120 ng/L, 98 ng/L, and 90 ng/L, respectively. Their maximum concentrations were 2200 ng/L, 2100 ng/L, 210 ng/L, 180 ng/L, and 250 ng/L, respectively. Albendazole, found in samples, is widely utilized to treat parasitic worm infections in both humans and animals (Ido et al., 2025). Rwanda, similarly to other sub-Saharan African countries, implements large-scale albendazole administration through school- and community-based mass deworming programmes, resulting in widespread and recurrent population exposure to albendazole (WHO, 2018). The average concentration of albendazole in this study (960 ng/L) was lower than the estimated surface water concentration in the East African region (20,000 ± 150 ng/L), calculated using a model based on consumption (Belew et al., 2021).

Five of the nine targeted pesticides and all four targeted polyphenols were detected. The average concentration of metalaxyl found in this study (70 ng/L) was within the range of 60 to 4800 ng/L reported for samples from Lake Muhazi, Lake Mugesera, and the Sebeya River in Rwanda (Houbraken et al., 2017). Metalaxyl has been detected in surface waters worldwide, with average concentrations ranging from 18 ng/L to 1300 ng/L (Struger et al., 2016; Zheng et al., 2016) consistent with observations in this study. Caffeic acid was the most abundant polyphenol, with an average concentration of 49 ng/L and a maximum concentration of 160 ng/L. Reported concentrations of polyphenols, including chlorogenic and ferulic acids, range from 60 ng/L to 24,000 ng/L (Chen et al., 2021; Ramos et al., 2021). Levels of polyphenols in aquatic environments depend on factors such as waste management, land use, seasonal runoff, and vegetation inputs (Ramos et al., 2021; Ziegler et al., 2025).

### Distribution of organic compounds in the Nyabugogo River

**Fig. 2:**
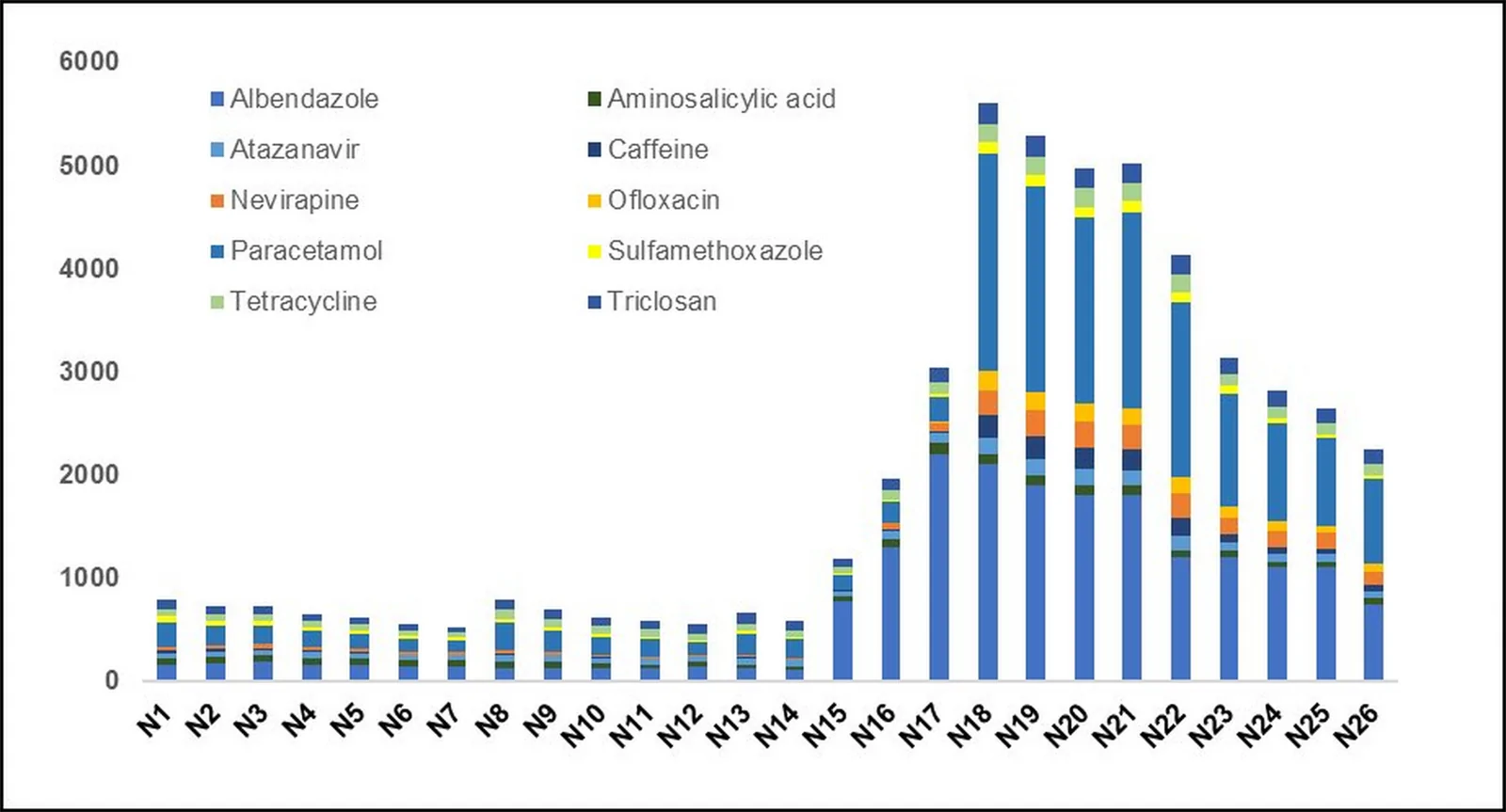
Distribution of the top ten pharmaceuticals (with higher concentrations in ng/L) across 26 sampling points in the Nyabugogo River (N1-N26)

Concentrations of pharmaceuticals in the Nyabugogo River showed a distinct rural–urban gradient, with consistently low levels in rural and peri-urban areas and markedly high levels in urban areas (Fig. 2). In addition, statistics indicated that the difference in average concentrations in the river between rural and peri-urban areas was not significant (*p* = 0.08), whereas the difference between rural and urban areas was significant (*p* = 0.017). The highest concentrations were recorded at sites N18–N21, which are reported to be the most polluted points in the Nyabugogo wetland (Sekomo et al., 2014). At the urban sites, the Nyabugogo River receives untreated domestic, institutional (healthcare and educational), and commercial effluents via channels C5-C8, as well as treated and untreated inputs from the Mpazi tributary (T9) and industrial discharges from Kigali. The contamination of rivers crossing the cities was also shown by Morin-Crini et al. (2022) in their review paper describing water pollution by emerging pollutants in a global context (Morin-Crini et al., 2022). Additional pollution sources may include untreated wastewater and sewage from the Nyabugogo food market (via C9), as reported in studies of the Nyabugogo wetland (Ministry of Environment, 2018); Musabyimana & Bazimenyera, 2023; Omara et al., 2020). No channels were identified between N21 and N26, and pharmaceutical concentrations declined along this reach, partly due to dilution by the Yanze tributary (T10) between N22 and N23, and the major degradation processes, photolysis, microbial degradation and sorption.

**Fig. 3:**
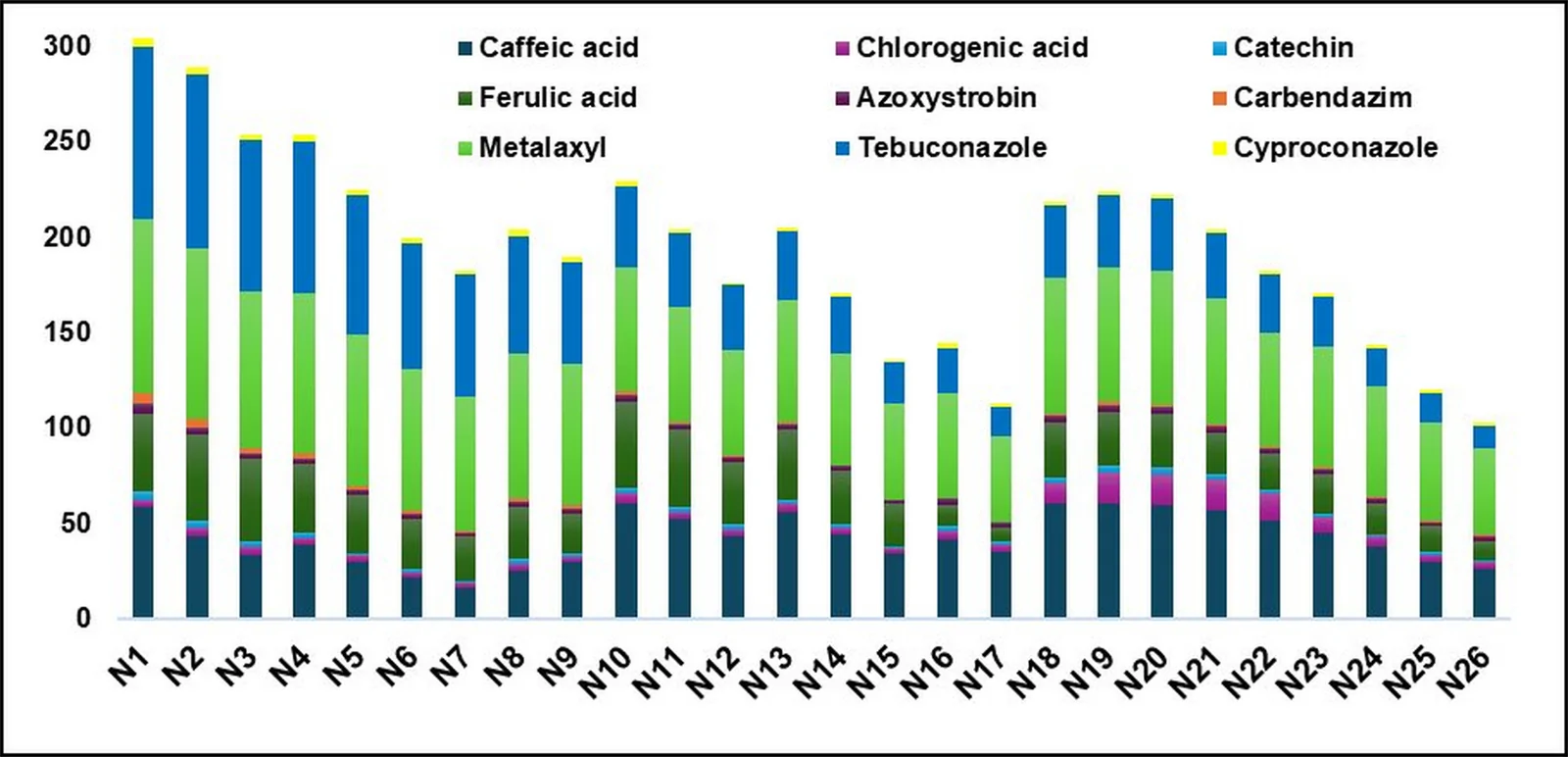
Distribution of pesticides and polyphenols across 26 sampling points in the Nyabugogo River (N1-N26)

The concentrations of pesticides and polyphenols measured in the Nyabugogo River across rural, peri-urban, and urban sites did not differ significantly (t-test, *p* = 0.99), as indicated in Fig. 3. Although total concentrations generally decreased downstream, substantial overlap among land-use categories was observed, indicating that the river functions as a hydrologically integrated system rather than discrete pollution zones (Gavrilaș et al., 2025). This homogenization may be caused by continuous inputs from multiple wastewater tributaries and channels discharging into the river (Anand et al., 2022; Gavrilaș et al., 2025; Xu et al., 2019). The fact that these discharges are scattered can increase variability within these three areas and obscure any significant differences expected between rural and peri-urban agricultural sources and urban runoff (Gavrilaș et al., 2025; Ziegler et al., 2025). Moreover, flow dynamics and degradation processes such as microbial breakdown and photolysis could further mix and normalize concentrations across the river continuum (Picó et al., 2020; Schwanen et al., 2023).

Pesticide occurrence in rural and peri-urban areas may be attributable to agricultural activities and runoff from cultivated fields, as reported by Narcisse et al., (2023) in their evaluation of stormwater runoff in the Nyabugogo River catchment (Narcisse et al., 2023). In urban areas, despite limited agricultural activity, elevated pesticide concentrations at sites N18–N20 may also result from wastewater generated during the washing of fruits and vegetables at the major food market at the Nyabugogo Centre. Additionally, the Mpazi tributary (T9) may also contribute by transporting wastewater from restaurants and abattoirs at the centre.

The presence of polyphenols in the Nyabugogo River is likely attributable to the degradation of fruit and vegetable waste entering the aquatic system (Ndwabu et al., 2023). In addition, effluents from industries, e.g., the Kabuye sugar factory, are discharged directly into the river as also observed by Edwin Byusa et al. (Edwin, 2013) and may substantially contribute to the elevated concentrations of caffeic and ferulic acids observed at sampling site N10 in the peri-urban zone, since sugarcane stems and leaves are known to contain significant levels of phenolic acids, particularly caffeic and ferulic acids (Lou et al., 2025).

### Concentration variability at the N21 sampling site in the Nyabugogo River

**Fig. 4:**
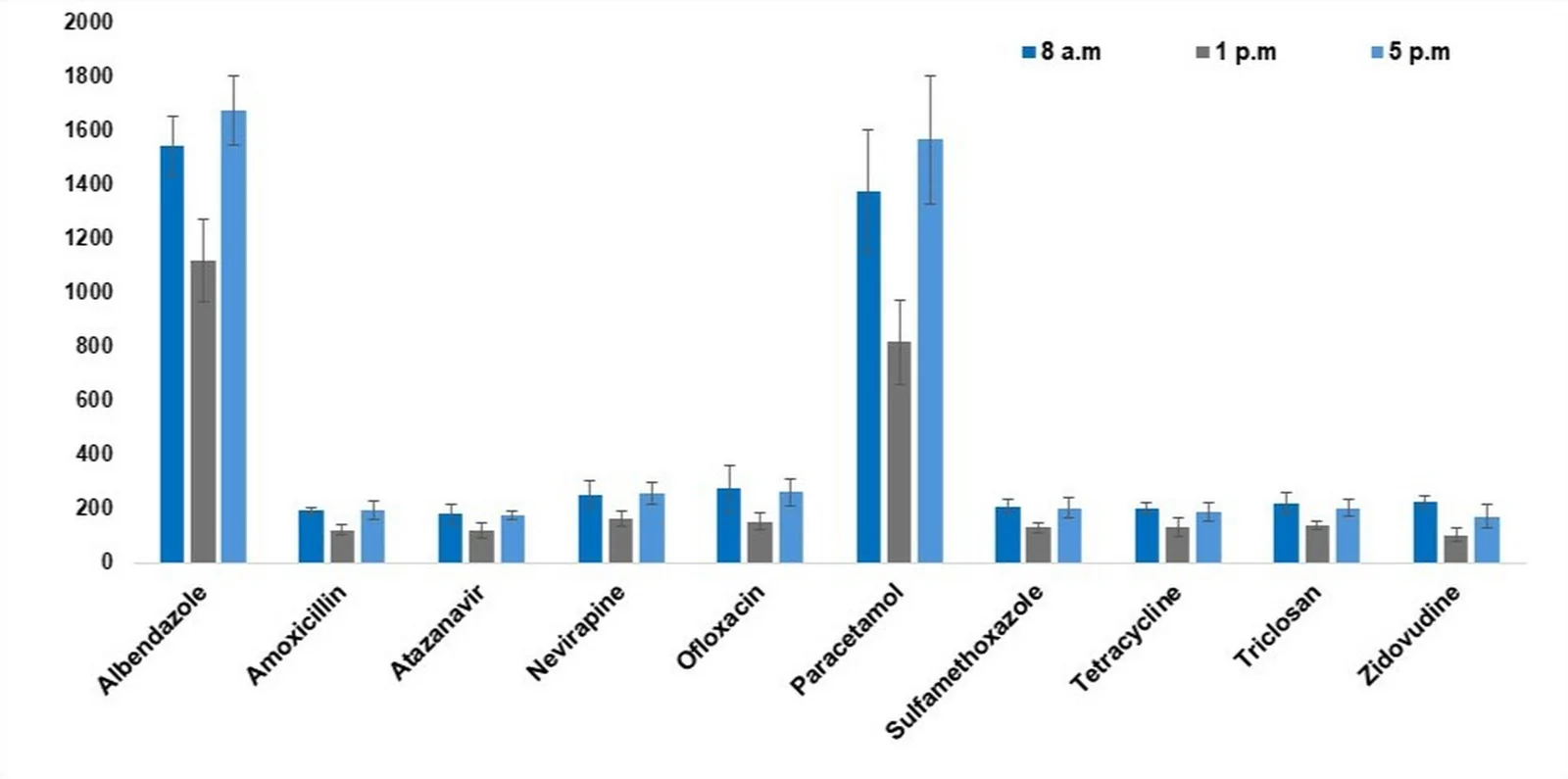
Comparison of average concentrations in ng/L (*n* = 5) of the ten pharmaceuticals detected at the sampling point N21 in the Nyabugogo River, with the highest levels at different times of day (8 a.m., 1 p.m., and 5 p.m.)

The investigation of variations in concentrations of selected compounds in water samples collected at site N21 showed that the average concentrations of detected pharmaceuticals over the five days ranged from < LOQ ng/L to 1400 ng/L, as indicated in Table S5 of the supplementary materials. Figure 4 showed that concentrations of pharmaceuticals at 8 a.m. and 5 p.m. were higher than at midday (1 p.m.), and the p-values from the ANOVA indicated that differences in concentrations for each analyte at these times of day were statistically significant (all p-values were lower than 0.05).

Variations in pharmaceutical concentrations observed at N21 in the Nyabugogo River (approximately 200 m from the Nyabugogo Centre) reflect both the daily patterns of domestic water use and the structural characteristics of Kigali’s sanitation and faecal sludge management systems. Elevated concentrations were detected at 8 a.m., with an average cumulative concentration of 7300 ng/L, compared with 3900 and 6000 ng/L at 1 p.m. and 5 p.m., respectively. According to what was reported in different articles on sanitation systems in Kigali, approximately 95% of households in Kigali rely on onsite sanitation systems, with pit latrines accounting for roughly 85% of these facilities, which can overflow or leak into adjacent channels (Akumuntu et al., 2017; Murebwayire et al., 2025; Rutayisire & Kuria, 2024). In addition, Kigali does not yet have a formal centralized sewer network; instead, it operates with 15 semi-centralized sewage treatment systems that only serve a small fraction of the urban population, leaving most wastewater and faecal sludge unmanaged or partially treated before entering the environment (Burt et al., 2019; Murebwayire et al., 2025; Rutayisire & Kuria, 2024; Tsinda et al., 2021).

In addition to these structural limitations, human behaviour related to faecal sludge disposal can also contribute, since there is limited access to affordable and reliable faecal sludge management services (Akumuntu et al., 2017; Burt et al., 2019; Murebwayire et al., 2025; Tsinda et al., 2021). Illegal faecal sludge dumping, driven by the high cost and limited capacity of formal emptying services in Kigali, has been reported (Akumuntu et al., 2017; Rutayisire & Kuria, 2024; Tsinda et al., 2021) and is typically carried out early in the morning or in the evening to minimize the risk of being caught and punished (Murebwayire et al., 2025; Tsinda et al., 2013, 2021). Moreover, informal channels, commonly found in upstream informal settlements, may accumulate wastewater overnight and then release it in a concentrated flush in the morning (Akumuntu et al., 2017; Murebwayire et al., 2025).

Results from the N21 sampling point also showed that the average levels of pesticides and polyphenols ranged from 1.3 ng/L to 40 ng/L for pesticides, and from 1.9 ng/L to 37 ng/L for polyphenols. Table S6 in the supplementary information indicates average concentrations of pharmaceuticals, pesticides, and polyphenols at the N21 point.

**Fig. 5:**
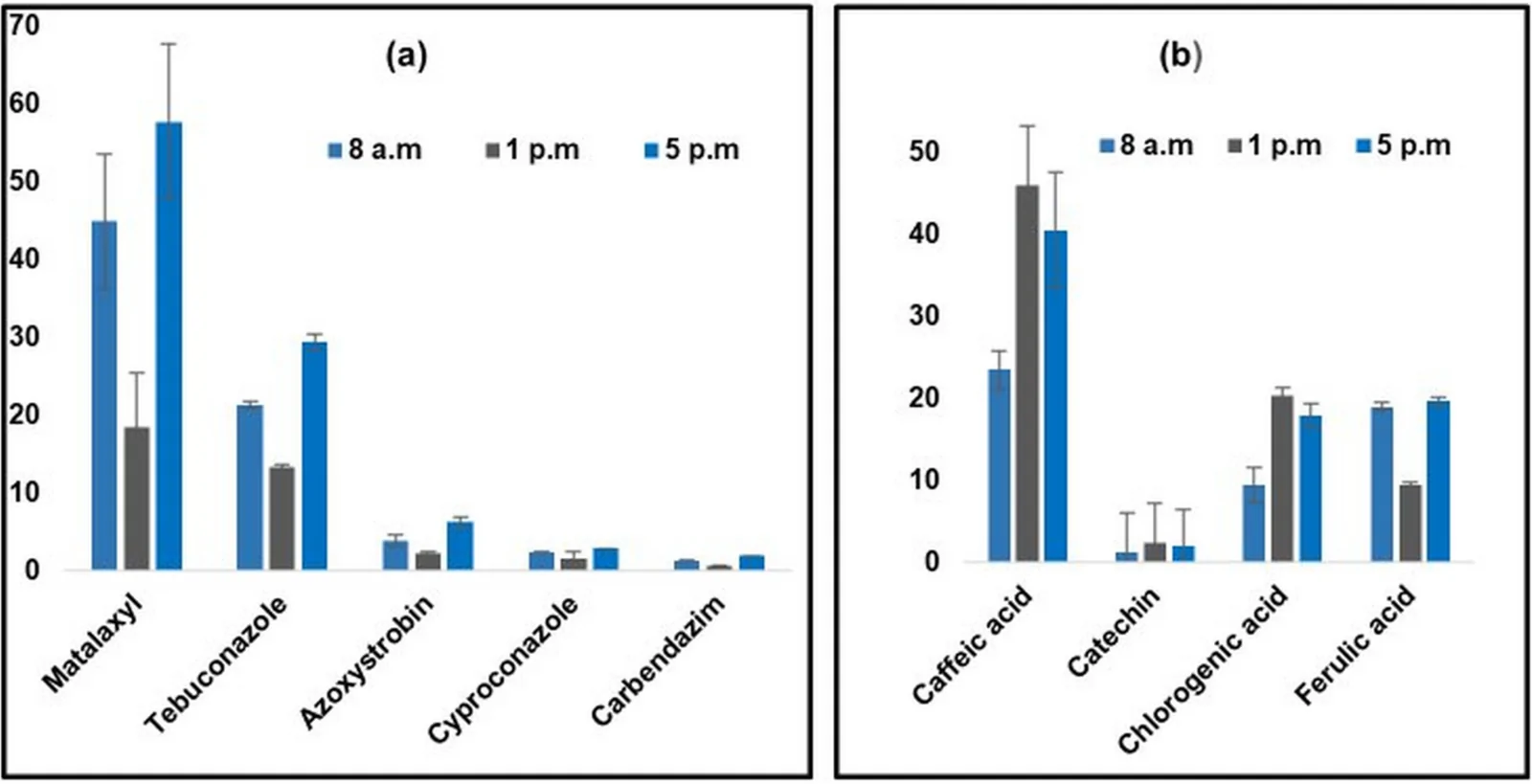
Comparison of average concentrations (ng/L) of pesticides (**a**) and polyphenols (**b**) detected and quantified at the N21 sampling point at different times of day (8 a.m., 1 p.m., and 5 p.m)

Also, as shown in Fig. 5a, concentrations at 8 a.m. and 5 p.m. were higher than those at 1 p.m. This could also be due to domestic wastewater discharges from Nyabugogo food market and restaurant cleanings, which often take place in the morning and evening. These cleaning patterns and their contribution to the contamination of water systems were also reported to occur in many cities worldwide (Morin-Crini et al., 2022; Wilkinson et al., 2022; Xu et al., 2019). The food market (Mutangana market) located at the Nyabugogo Centre is one of the main markets in Kigali, selling fruits and vegetables. When fruits and vegetables are washed at processing plants or in agricultural settings, the pesticides on their surfaces are washed into the water used (Nguyen et al., 2020; Sayed et al., 2021). Moreover, non-edible fractions of fruits and vegetables, including peels, skins, and leaves, are known to retain measurable pesticide residues (Nguyen et al., 2020; Sayed et al., 2021). During processing or disposal of these plant materials, the resulting wastewater can therefore serve as a pathway for the release of pesticide contaminants into the surrounding environment (Nguyen et al., 2020; Nuruzzaman et al., 2025).

Caffeic acid and chlorogenic acid showed a different trend, with higher concentrations observed at midday (Fig. 5b). These elevated concentrations may be linked to wastewater discharges occurring during industrial working hours (8 a.m.–5 p.m.). Effluent discharged from industries located in the Kigali Special Economic Zone (KSEZ) can reach the N21 sampling point in the Nyabugogo River after approximately one hour. KSEZ hosts over 100 industries, including coffee bean roasting facilities.

P-values from ANOVA indicated that the concentration variations between days for pharmaceuticals, pesticides, and polyphenols were not statistically significant, suggesting that the sources of contamination were pseudo-persistent, i.e. released at more or the same rate as they are degraded (see Table S7 of the supplementary information).

These findings demonstrate how both infrastructural challenges and socially mediated disposal practices influence the temporal variability of pharmaceutical pollution in the Nyabugogo River. They also underscore the need for strengthened faecal sludge management regulation, enhanced wastewater treatment capabilities, and enforcement strategies to mitigate pollutant loadings and protect water quality in rapidly urbanizing locations such as Kigali.

## Limitations of the study and its contribution

Some limitations should be considered when interpreting the findings of this study. As this study is the first investigation of pharmaceuticals, pesticides, and polyphenols in the Nyabugogo River and, more broadly, in Rwanda, comparable scientific studies from Rwanda and the region were limited. This constrained direct comparison of some of the observed concentrations and patterns with previously reported findings. Nevertheless, the results provide an important baseline for future investigations.

River discharge and flow rates in the tributaries and drainage channels were not measured concurrently with contaminant concentrations. Therefore, the observed concentration differences, particularly the morning and evening elevations at N21, cannot be unequivocally attributed to changes in contaminant loading, as variations in river flow and dilution may also have contributed. In addition, although the spatial distribution of contaminants and surrounding land-use characteristics provided indications of potential sources, direct source attribution was not possible. Domestic, institutional, commercial, industrial, agricultural, and market-related discharges were not individually characterized or quantified.

Despite these limitations, this study provides an important baseline for understanding organic chemical contamination in the Nyabugogo River and demonstrates the value of combining spatial surveys with within-day monitoring. The findings provide a foundation for seasonal and source-oriented investigations, including concurrent measurements of river discharge and contaminant loads, and for developing evidence-based strategies to reduce inputs of organic contaminants into the river.

## Conclusion

The contamination of Rwandan rivers by pharmaceuticals, pesticides, and polyphenols is a critical issue that demands attention from all stakeholders. This study detected 66 chemicals in the Nyabugogo River, with the highest average concentrations for pharmaceuticals (albendazole, 960 ng/L), pesticides (metalaxyl, 70 ng/L), and polyphenols (caffeic acid, 49 ng/L). The highest pharmaceutical concentrations were observed in water samples from urban areas. Pesticide and polyphenol concentrations did not differ significantly across rural, peri-urban, and urban areas, suggesting that agricultural activities in rural and peri-urban areas may substantially contribute to contamination of the Nyabugogo River far downstream. Analysis of concentration changes at sampling point N21 near the Nyabugogo Centre showed that within-day differences in all analysed pharmaceuticals and pesticides were statistically significant. However, day-to-day variations were not statistically significant, indicating that contamination sources are likely pseudo-persistent. The findings showed that it is necessary to improve the management of wastewater and fecal sludge, enhance the treatment of domestic and industrial effluents, reduce runoff of agricultural and market-related pollutants, and establish regular monitoring of organic contaminants. Future research should extend monitoring to both the wet and dry seasons, examine sediments and transformation products, and assess mixture toxicity and ecological risk. Through such comprehensive monitoring, a more solid understanding of the long-term environmental effects of these organic contaminants can be achieved, and effective strategies for protecting the Nyabugogo River and the Nyabarongo—Akagera—Nile river system can be developed.

## Supplementary Information

Below is the link to the electronic supplementary material.

Supplementary file1 (PDF 101 KB)

Supplementary file2 (PDF 58 KB)

Supplementary file3 (PDF 395 KB)

Supplementary file4 (PDF 178 KB)

Supplementary file5 (PDF 54 KB)

Supplementary file6 (PDF 87.1 KB)

Supplementary file7 (XLSX 26 KB)

## Data Availability

No datasets were generated or analysed during the current study.
