# Supplementary material for: Occurrence and concentration variability of pharmaceuticals, pesticides, and polyphenols in Nyabugogo River, Rwanda
Source: Environ Monit Assess. 2026 Oct 15;198(11):1196. doi: 10.1007/s10661-026-16028-z (PMC13672450; doi:10.1007/s10661-026-16028-z)
Supplement: Supplementary file 1 [file 10661_2026_16028_MOESM1_ESM.pdf]

Table S1. Sampling sites and geographical coordinates. **T** represents tributary, **N** are samples from the river, and **C** represents channels and informal drainages

**S. P**	**Site names**	**Sampling date/time**	**Coordinates**	
T1	Muyanza	09/12/2024:9h00 AM	1°46'15"S	30°07'21"E
T2	Cyamutara	09/12/2024:9h20 AM	1°46'40"S	30°07'49"E
T3	Muhazi lake	09/12/2024:9h350 AM	1°46'46"S	30°08'19"E
N1	Joint point Muhazi -Muyanza Cyamutara	09/12/2024:9h52 AM	1°47'06"S	30°07'57"E
T4	Kajevuba tributary	09/12/2024:10h12 AM	1°47'39"S	30°07'47"E
N2	Cyamarita	09/12/2024:10h28 AM	1°48'32"S	30$_o$07'41"E
T5	Rusine tributary	09/12/2024:10h56 AM	1°48'53"S	30°05'50"E
N3	Nyabugogo Rusine	09/12/2024:11h16 AM	1°48'54"S	30o05'51"E
T6	Marenge centre	09/12/2024:11h50 AM	1°49'34"S	30°04'46"E
N4	Marenge -Nyange 1	09/12/2024:11h50 AM	1°50'14"S	30°04'57" E
N5	Nyange /Quarrying (stones-sand)	10/12/2024:09h50 AM	1°50'27"S	30°05'10" E
N6	Jabana Mukobwa mwiza	10/12/2024:10h22 AM	1°50'32"S	30°05'17" E
N7	Nyacyonga Strabag near centre	10/12/2024:10h37 AM	1°51'34"S	30°05'42" E
T7	Nyacyonga	10/12/2024:10h54 AM	1°52'01"S	30°05'18"E
N 8	Nyacyonga centre- Bridge	10/12/2024:11h38 AM	1°51'58"S	30°05'15"E
C1	Jabana canal from rice plantation	10/12/2024:12h05 PM	1°52'58"S	30°04'38"E
N9	Jabana near rice plantation	10/12/2024:12h25 PM	1°52'59"S	30°04'38"E
C2	Kabuye sugar works factory channel	10/12/2024:12h55 PM	1°53'30"S	30°04'12"E
N10	Nyabugogo (100m from Kabuye Factory)	10/12/2024:13h07 PM	1°53'31"S	30°04'09"E
N11	Jabana ku mavoka	11/12/2024:09h49 AM	1°53'42"S	30°03'49"E
N12	Gisozi bridge	11/12/2024:10h16 AM		
T8	Karuruma river	11/12/2024:10h26 AM	1°54'08"S	30°03'03"E
N13	Nyabugogo+Karuruma river	11/12/2024:10h44 AM	1°54'21"S	30°03'08"E
C3	Gatsata- Prowater channel	11/12/2024:11h25 AM	1°54'48"S	30°02'57"E
N14	Nyabugogo+ water from Prowater	11/12/2024:11h46 AM	1°54'50"s	30°02'59"E
C4	Channel from Rubis Station	11/12/2024:12h09 AM	1°55'03"S	30°02'54"E
N15	Gatsata near Rubis petrol station	11/12/2024:12h25 PM	1°55'03"S	30°02'58"E
C5	Akamwunguzi channel 1	11/12/2024:12h54 PM	1°55'32"S	30°02'54"E
C6	Akamwunguzi channel 2	11/12/2024:13h00 PM	1°55'32"S	30°02'51"E
C7	Akamwunguzi channel 3	11/12/2024:13h10 PM	1°55'30"S	30°02'51"E
N16	Nyabugo Rwantexco bridge	12/12/2024:09h05 AM	1°55'58"S	30°02'57"E
C8	Channel from Gatsata garage	12/12/2024:09h46 AM	1°56'18"S	30°02'44"E
N17	Nyabugogo between garage & Bridge	12/12/2024:09h57 AM	1°56'17"S	30°02'40"E
T9	Mpazi tributary	12/12/2024:10h20 AM	1°56'23"S	30°02'41"E
C9	Channel from food market	12/12/2024:10h34 AM	1°56'20"S	30°02'42"E
N18	Nyabugogo Gatsata Bridge 2	12/12/2024:11h10 AM	1°56'21"S	30°02'35"E
N19	Nyabugogo Bridge near Car park	12/12/2024:11h27 AM	1°56'21"S	30°02'35"E
N20	Nyabugogo near Gatsata shops	12/12/2024:11h50 AM		
N21	Gatsata Nyabugogo at 100 m of bridge	12/12/2024:12h20 AM	1°56'25"S	30°02'34"E
N22	Nyabugogo Discentre	13/12/2024: 09h00 AM	1°56'22"S	30°02'37"E

T10	Yanze river	13/12/2024:	09h25AM	1°56'34''S	30°02'03''E
N23	Nyabugogo + Yanze River water	13/12/2024:	09h34 AM	1°56'36''S	30°02'01''E
N24	Riverside Motel(Arsenal)	13/12/2024:	10h15 AM	1°56'49''S	30°01'14''E
N25	Nyabugogo/ Nzove	13/12/2024:	10h50 AM	1°56'53''S	30°01'04''E
N26	Nyabugogo/Nyabarongo	13/12/2024:	11h30 AM	1°57'17''S	30°00'24''E

26 samples from the river, 10 samples from tributaries and 9 samples from channels and drainages

S.P: sampling point
