# Supplementary material for: Occurrence and concentration variability of pharmaceuticals, pesticides, and polyphenols in Nyabugogo River, Rwanda
Source: Environ Monit Assess. 2026 Oct 15;198(11):1196. doi: 10.1007/s10661-026-16028-z (PMC13672450; doi:10.1007/s10661-026-16028-z)
Supplement: Supplementary file 2 [file 10661_2026_16028_MOESM2_ESM.pdf]

Table S2. Transitions, precursors, and retention times of pesticides and polyphenols (standards and internal standards) considered in this study

Compound	Start Time (min)	End Time (min)	Polarity	Precursor (m/z)	Product (m/z)	Collision Energy (V)
Azoxystrobin	0	15	Positive	404.12	344.076	27
Azoxystrobin	0	15	Positive	404.12	372.067	16
Caffeic acid	0	15	Negative	179	107.058	21.9
Caffeic acid	0	15	Negative	179	135	15.1
Carbamazepin D10 Qual	0	15	Positive	247.143	202.111	34.32
Carbamazepin D10 Quan	0	15	Positive	247.143	204.111	21.073
Carbendazim	0	15	Positive	192.155	132.151	33
Carbendazim	0	15	Positive	192.155	160.13	20
Catechin	0	15	Positive	291	123.058	15.7
Catechin	0	15	Positive	291	139	15.7
Chlorogenic acid	0	15	Negative	353.07	179	15
Chlorogenic acid	0	15	Negative	353.07	191	16.5
Chlorpyrifos D10	0	15	Positive	361.939	200.889	20
Chlorpyrifos D10	0	15	Positive	361.939	329.889	10
Cyproconazole	0	15	Positive	292.17	70.326	22
Cyproconazole	0	15	Positive	292.17	125.121	30
Ferulic acid	0	15	Negative	193	134	15.7
Ferulic acid	0	15	Negative	193	178	12.6
Fluconalzole IS	0	15	Positive	311.096	222.986	18
Fluconalzole IS	0	15	Positive	311.096	242.058	16
Metalaxyl	0	15	Positive	280.2	160.172	26
Metalaxyl	0	15	Positive	280.2	220.128	16
Tebuconazole	0	15	Positive	308.2	70.338	24
Tebuconazole	0	15	Positive	308.2	125.106	35
