# Supplementary material for: Occurrence and concentration variability of pharmaceuticals, pesticides, and polyphenols in Nyabugogo River, Rwanda
Source: Environ Monit Assess. 2026 Oct 15;198(11):1196. doi: 10.1007/s10661-026-16028-z (PMC13672450; doi:10.1007/s10661-026-16028-z)
Supplement: Supplementary file 3 [file 10661_2026_16028_MOESM3_ESM.pdf]

Retention times (RT), Limit of quantification (LOQ), Internal standard (IS) used for each analyte, the coefficient of determination (R^2) of the calibration curve, recoveries (%), and Relative standard Deviation (RSD) calculated after performing laboratory controls (n=6).

	Analyte	RT (min)	LOQ (ng/L)	IS	R^2	Recoveries (%)	RSD (%)
1	Abacavir	1.01	2	Ofloxacin_D3	0.997	99.3	8.4
2	Aciclovir	1.02	0.1	Tramadol_IS	0.996	87.2	10.7
3	Albendazole	6.97	2	Flecainide_D4	0.997	103.5	4.1
4	Aminosalicylic acid	1.86	0.5	Tramadol_IS	0.998	100	6.7
5	Amlodipine	7.41	1	Oxazepam_IS	0.997	97.8	10.4
6	Amoxicillin	4.01	5	Ofloxacin_D3	0.998	99.1	10.3
7	Atazanavir	8.81	7	Flecainide_D4	0.999	89.7	6.1
8	Atenolol	1.02	5	Fluconazole_IS	0.999	94.2	5.7
9	Caffeine	4.38	10	Tramadol_IS	0.997	100,0	3.7
10	Carbamazepine	5.98	1	Carbamazepine_D10	0.999	102,3	5.6
11	Cetirizine	7.72	5	Oxazepam_IS	0.999	92.8	7.7
12	Chlorpheniramine	5.25	1	Tramadol_IS	0.997	87.3	9.2
13	Ciprofloxacin	4.41	10	Ciprofloxacin_D8	0.999	92.1	10.2
14	Clarithromycin	7.66	1	Oxazepam_IS	0.999	99.4	4.7
15	Clotrimazole	7.43	1	Clotrimazole_IS	0.996	88.3	9.8
16	Cloxacillin	7.92	1	Risperidone_IS	0.998	90.6	3.3
17	Darunavir	7.77	5	Oxazepam_IS	0.998	90.3	5.3
18	Dexamethazone	7.8	0.5	Oxazepam_IS	0.997	102.2	11.3
19	Diazepam	8.25	4	Oxazepam_IS	0.998	95.9	7.2
20	Diclofenac	9.01	2	Diclofenac_IS	0.998	93.9	5.2
21	Diltiazem	6.59	0.5	Risperidone_IS	0.999	87.5	6.6
22	Efavirenz	9.3	2	Oxazepam_IS	0.998	88.7	10.3
23	Ethambutol	1.02	1	Fluconazole_IS	0.997	91.1	7.8
24	Fexofenadine	7.13	0.1	Oxazepam_IS	0.998	90.4	8.8
25	Haloperidol	6.38	0.1	Risperidone_IS	0.999	98.3	11.1
26	Ibuprofen	1.06	5	Trimetropim_D9	0.993	99.5	4.4
27	Irbesartan	7.95	0.5	Oxazepam_IS	0.999	100.3	4.8
28	Isoniazid	1.19	2	Tramadol_IS	0.994	85.6	5.5
29	Ketamine	7.41	0.1	Oxazepam_IS	0.995	93.4	9.1
30	Lamivudine	1.04	5	Ofloxacin_IS	0.996	99.9	4.7
31	Levofloxacin	4.33	0.1	Ofloxacin_IS	0.998	99.8	3.8
32	Lidocaine	1.06	0.1	Tramadol_IS	0.998	98.3	7.9
33	Loperamide	7.53	0.5	Oxazepam_IS	0.996	88.5	10.2
34	Lorazepam	7.82	0.1	Oxazepam_IS	0.998	90.4	8.4
35	Mefenamic acid	9.19	0.1	Diclofenac_IS	0.995	93.5	6.9
36	Methyldopa	1.02	0.1	Fluconazole_IS	0.995	97.7	6.3
37	Miconazole	7.92	1	Oxazepam_IS	0.995	84.8	9.6
38	Moxifloxacin	5.44	0.5	Ofloxacin_D3	0.999	99.2	5.5

39	Nalidixic acid	6.68	0.1	Risperidone_IS	0.995	101.8	7.6
40	Naproxen	8.19	2	Oxazepam_IS	0.996	98.3	8.3
41	Nevirapine	5.87	5	Risperidone_IS	0.997	99.4	6.3
42	Nifedipine	7.47	1	Flecainide_D4	0.998	96.4	7.1
43	Ofloxacin	4.33	4	Ofloxacin_D3	0.993	102.1	9.4
44	Omeprazole	5.85	1	Fluconazole_IS	0.999	94.8	7.5
45	Oxazepam	7.68	0.5	Oxazepam_IS	0.997	98.8	6.4
46	Oxytetracycline	4.25	2	Ofloxacin_D3	0.996	99	5.7
47	Paracetamol	1.05	10	Tramadol_IS	0.999	97.4	7.9
48	Promethazine	6.67	1	Risperidone_IS	0.994	93.4	7.3
49	Propranolol	5.92	1	Tramadol_IS	0.997	98.3	4.8
50	Pyrazinamide	1.01	0.1	Fluconazole_IS	0.995	102.5	9.7
51	Sulfadiazine	1.01	0.1	Tramadol_IS	0.995	99.3	9
52	Sulfamethoxazole	4.34	5	Fluconazole_IS	0.996	95.6	7.3
53	Telmisartan	7.7	0.1	Oxazepam_IS	0.993	99.9	6.4
54	Terbutaline	1.01	2	Tramadol_IS	0.991	99.1	8.3
55	Tetracycline	4.29	5	Ofloxacin_D3	0.999	98.6	7.4
56	Triclosan	7.77	20	Flecainide_D4	0.999	100.4	9.9
57	Zidovudine	4.49	10	Risperidone_IS	0.998	99.6	8.6
58	Azoxystrobin	6.93	0.5	Carbamazepine_13C	0.996	99.1	9.7
59	Caffeic acid	7.11	5	Fluconazole_IS	0.999	97.3	2.9
60	Carbendazim	3.13	0.1	Fluconazole_IS	0.999	101.4	7.5
61	Catechin	3.60	0.1	Fluconazole_IS	0.997	98.7	8.1
62	Chlorogenic acid	3.36	0.5	Fluconazole_IS	0.992	88.2	13.8
63	Cyproconazole	7.22	0.5	Carbamazepine_13C	0.994	89.9	7.3
64	Ferulic acid	4.25	2	Carbamazepine_13C	0.998	107.1	9.2
65	Metalaxyl	6.30	5	Carbamazepine_13C	0.998	100.2	6.6
66	Tebuconazole	7.81	5	Carbamazepine_13C	0.996	99.6	9.6

:
