# Supplementary material for: Occurrence and concentration variability of pharmaceuticals, pesticides, and polyphenols in Nyabugogo River, Rwanda
Source: Environ Monit Assess. 2026 Oct 15;198(11):1196. doi: 10.1007/s10661-026-16028-z (PMC13672450; doi:10.1007/s10661-026-16028-z)
Supplement: Supplementary file 4 [file 10661_2026_16028_MOESM4_ESM.pdf]

Table S4 Average concentrations of detected and quantified pharmaceuticals in Nyabugogo River at all sampling points (n=45)

Pharmaceutical	Therapeutic group	ATC code	LOQ (ng/L)	Max (ng/L)	Min (ng/L)	Average (ng/L)	Frequency* (%)
Abacavir	Antiretroviral	J05AF06	2	432.5	2.7	61.3	91
Aciclovir	Antiretroviral	J05AB01, S01AD03	0.1	54.8	0.1	5.4	89
Albendazole	Antihelminthic	P02CA03	2	4184.6	51.4	965.9	93
Aminosalicylic acid	Antimicobacterial	J04AA01	0.5	200.4	2.8	76.5	98
Amlodipine besylate	Calcium blocker	C08CA01	1	72.6	1.8	14.4	100
Amoxicillin	Antibacterial	J01CA04	5	246.2	<LOQ	54.9	93
Atazanavir	Antiretroviral	J05AE08	7	337.3	10.3	99.8	98
Atenolol	Beta Blocker	C07AB03	5	99.8	5.7	13.1	98
Caffeine	Psychoanaleptic	N06BC01	10	527.5	<LOQ	72.3	100
Carbamazepine	Antiepileptic	N03AF01	1	28.3	<LOQ	5.4	100
Cetirizine	Antihistaminic	R06AE07	0.5	9.3	<LOQ	1.8	96
Chlorpheniramine	Antihistaminic	R06AB04	1	33.9	<LOQ	3.8	98
Ciprofloxacin	Antibacterial	J01MA02	10	432.9	<LOQ	50.7	98
Clarithromycin	Antibacterial	J01FA09	1	653.4	3.2	89.8	87
Clotrimazole	Stomatological drug	A01AB18, D01AC01, G01AF02	1	215.2	1.8	34.9	80
Cloxacillin	Antibacterial	J01CF02	1	358.1	2.2	60.1	96
Darunavir	Antiretroviral	J05AE10	0.5	15.7	0.5	1.8	84
Dexamethazone	Stomatological drug	A01AC02, C05AA09, D07AB19	0.5	78.5	1.7	12.5	98
Diazepam	Psycholeptic	N05BA01	0.5	39.3	<LOQ	6.9	96
Diclofenac	Antiinflammatory	M01AB05, M02AA15, S01BC03	2	62.7	<LOQ	10.3	98
Diltiazem	Calcium blocker	C08DB01	0.5	3.1	<LOQ	0.7	93
Efavirenz	Antiretroviral	J05AG03	2	264.9	<LOQ	40.8	96
Ethambutol hydrochloride	Antimicobacterial	J04AM03	1	196	<LOQ1	37.2	98
Fexofenadine	Antihistaminic	R06AX26	0.1	34.8	<LOQ	4.2	89
Haloperidol	Psycholeptic	N05AD01	0.1	0.7	<LOQ	0.2	91
Ibuprofen	Antiinflammatory	M01AE01, M02AA13, G02CC01	5	86.4	5.1	22.6	98
Irbesartan	Angiotensive	C09CA04	0.5	122.8	1.4	20.3	93

Isoniazid	Antimicobacterial	J04AC01	2	249.7	2.4	30.4	98
Ketamine	Anesthetic	N01AX03	0.1	8.3	0.1	0.9	87
Lamivudine	Antiretroviral	J05AF05	5	418.5	<LOQ	48.6	96
Levofloxacin	Antibacterial	J01MA12	0.1	35.4	<LOQ	4.8	98
Lidocaine	Cardiac therapy	C01BB01, C05AD01, D04AB01	0.1	0.5	0.1	0.2	69
Loperamide	Antidiarrheal	A07DA03	0.5	262	0.5	64.8	100
Lorazepam	Psycholeptic	N05BA06	0.1	0.9	<LOQ	0.2	87
Mefenamic acid	Antiinflammatory	M01AG01	0.1	13.9	0.1	2.7	98
Methyldopa	Antihyprtensive	C02AB01 C02LB01 C02AB02	0.1	8.6	<LOQ	1.1	91
Miconazole	Antifungal	D01AC02 , A01AB09, G01AF04	1	59.5	<LOQ	12.4	96
Moxifloxacin	Antibacterial	J01MA14	0.5	21.5	0.6	3.8	100
Nalidixic acid	Antibacterial	J01MB02	0.1	5.6	<LOQ	0.9	91
Naproxen	Antiinflammatory	M01AE02, M02AA12, G02CC02	2	48.2	2.8	6.7	98
Nevirapine	Antiretroviral	J05AG01	5	989.2	5.1	164.9	98
Nifedipine	Calcium blocker	C08CA05	1	4.5	<LOQ	1.4	89
Ofloxacin	Antibacterial	J01MA01, S01AX11	4	544.6	<LOQ	65.0	93
Omeprazole	Antacid	A02BC01	1	52.4	1.3	13.4	93
Oxazepam	Psycholeptic	N05BA04	0.1	1.3	<LOQ	0.3	98
Oxytetracycline	Antibacterial	J01AA06, S01AA04 G01AA07	2	5.7	<LOQ	2.3	93
Paracetamol	Analgesic	N02BE01	10	5961.3	15.2	787.0	98
Promethazine	Antihistaminic	R06AD02, D04AA10	0.1	0.6	<LOQ	0.2	67
Propranolol	Beta Blocker	C07AA05	0.1	2.1	<LOQ	0.4	89
Pyrazinamide	Antimicobacterial	J04AK01	0.1	58.5	0.1	9.3	96
Sulfadiazine	Antibacterial	J01EC02	0.1	0.9	<LOQ	0.1	96
Sulfamethoxazole	Antibacterial	J01EC01	5	295.2	5.7	61.3	98
Telmisartan	Angiotensive	C09CA07	0.1	3.4	0.1	0.7	91
Terbutaline	Obstructive airway	R03AC03	1	13.2	1.4	3.1	93
Tetracycline	Antibacterial	J01AA07, A01AB13, D06AA04	5	369.8	19.9	124.1	96
Triclosan	Medicated dressing	D09AA06	20	431.9	21.0	141.7	100
Zidovudine	Antiretroviral	J05AF01	10	777.2	16.7	91.5	100

**Frequencies were calculated based on LOQs in this study*
