# Supplementary material for: Occurrence and concentration variability of pharmaceuticals, pesticides, and polyphenols in Nyabugogo River, Rwanda
Source: Environ Monit Assess. 2026 Oct 15;198(11):1196. doi: 10.1007/s10661-026-16028-z (PMC13672450; doi:10.1007/s10661-026-16028-z)
Supplement: Supplementary file 5 [file 10661_2026_16028_MOESM5_ESM.pdf]

Table S5 Average concentrations of detected and quantified pesticides and polyphenols in the Nyabugogo River at all sampling points (n=45)

Chemical	Use	LOQ (ng/L)	Min (ng/L)	Max (ng/L)	Median (ng/)	Average (ng/L)	Frequency* (%)
**Azoxystrobin**	Fungicide	0.5	<LOQ	19.1	3.1	3.7	98
**Caffeic acid**	Polyphenol	5	12.5	160.2	43.7	49.1	100
**Carbendazim**	Fungicide	0.1	<LOQ	11.4	1.5	1.9	89
**Catechin**	polyphenol	0.1	<LOQ	40.7	2.3	3.6	100
**Chlorogenic acid**	polyphenol	0.5	0.5	92.1	4.6	11.4	100
**Cyproconazole**	Fungicide	0.5	<LOQ	6.2	2.3	2.7	96
**Ferulic acid**	Polyphenol	2	<LOQ	194.3	28.4	32.4	98
**Metalaxyl**	Fungicide	5	5.5	126.8	70.6	69.6	100
**Tebuconazole**	Fungicide	5	<LOQ	103.6	39.2	49.5	96

* *Frequencies were calculated based on LOQ*
