# Supplementary material for: Occurrence and concentration variability of pharmaceuticals, pesticides, and polyphenols in Nyabugogo River, Rwanda
Source: Environ Monit Assess. 2026 Oct 15;198(11):1196. doi: 10.1007/s10661-026-16028-z (PMC13672450; doi:10.1007/s10661-026-16028-z)
Supplement: Supplementary file 6 [file 10661_2026_16028_MOESM6_ESM.pdf]

Table S6. Average concentrations (ng/L)of detected and quantified pharmaceuticals at sampling point N21 of the Nyabugogo River at different times of the day and for 5 consecutive days and their relative standard deviation (RSD)

**Chemical**	**8:AM**	**1: PM**	**5:PM**	**8:AM**	**1: PM**	**5:PM**
		**Averages**			**RSD**	
Abacavir	165	102.6	156.4	14	20	22
Aciclovir	12	8.6	11.5	18	15	25
Albendazole	1545.1	1120.8	1677.3	16	21	20
Aminosalicylic acid	110.2	70.6	109.5	16	28	20
Amlodipine	20.5	12.7	17.8	20	16	20
Amoxicillin	192	122.2	193.2	6	17	18
Atazanavir	184	119.6	175.6	18	14	9
Atenolol	19.2	9.7	17.2	16	1	17
Azoxystrobin	3.9	2.1	6.2	5	18	14
Caffeic acid	23.5	46	40.5	17	14	13
Caffeine	148.5	73.2	130.7	21	15	17
Carbamazepine	21	15.5	21.5	14	2	16
Carbendazim	1.3	0.6	1.9	7	11	12
Catechin	1.3	2.4	2	16	15	12
Cetirizine	2.7	1.7	2.5	9	17	5
Chlorogenic acid	9.4	20.3	17.9	24	26	20
Chlorpheniramine	17.9	9.9	16.9	15	13	13
Ciprofloxacin	119.4	80.2	112.2	6	18	19
Clarithromycin	205.7	96.3	186.9	18	11	7
Clotrimazole	158.9	97.6	140.4	20	13	13
Cloxacillin	135.7	67	121.2	16	24	14
Cyproconazole	2.4	1.5	2.8	20	21	18
Darunavir	6.2	3.6	5.5	16	16	15
Dexamethazone	21.4	14.5	19.5	13	15	15
Diazepam	25.5	15	22.8	17	7	14
Diclofenac	32.6	17.2	30.7	18	13	16
Diltiazem	1.4	0.7	1.3	14	12	1
Efavirenz	131.6	72.4	139	22	13	22
Ethambutol	129.8	67.3	126.2	19	8	22
Ferulic acid	18.9	9.4	19.6	15	11	12
Fexofenadine	20	8.7	18.4	18	14	11
Haloperidol	0.7	0.5	0.7	15	23	24
Ibuprofen	52.4	38.2	48.3	18	12	9
Irbesartan	78.7	40.8	65.8	19	19	15
Isoniazid	104.7	71.7	100.4	16	18	14
Ketamine	4.7	2.3	3.7	25	25	27
Lamivudine	30.5	15.9	29.1	25	26	43
Levofloxacin	18.2	11.1	15.5	19	19	20
Lidocaine	0.1	0	0.1	9	10	17
Loperamide	117.6	63.7	113	13	19	14
Lorazepam	1.2	0.5	1	20	16	18

Matalaxyl	44.9	18.5	57.7	17	20	9
Mefenamic acid	7.3	5.8	6.3	14	22	12
Methyldopa	6.6	4.7	6.5	11	23	15
Miconazole	27.5	21.6	27.3	13	22	17
Moxifloxacin	18.9	9.6	15.8	22	8	16
Nalidixic acid	2.2	0.7	2	23	20	15
Naproxen	22.4	10.2	18.9	20	12	17
Nevirapine	252.6	164.5	257.2	20	16	15
Nifedipine	2.2	1.5	2.5	21	11	17
Ofloxacin	275.1	152.7	261.7	12	21	19
Omeprazole	21.1	13.2	18.6	19	19	21
Oxazepam	1.6	0.8	1.7	26	23	16
Oxytetracycline	3.5	2.5	3	17	21	19
Paracetamol	1375.8	818.4	1568.9	8	3	3
Promethazine	0.6	0.1	0.4	13	17	13
Propranolol	2.2	1.5	2.4	15	10	18
Pyrazinamide	32.7	23.6	32.1	12	15	16
Sulfadiazine	0.5	0.3	0.3	23	29	19
Sulfamethoxazole	209.4	129.2	203.7	12	14	18
Tebuconazole	21.2	13.3	29.4	15	14	16
Telmisartan	3.1	1.7	2.7	9	14	14
Terbutaline	7.1	4.2	4.5	21	28	25
Tetracycline	199.5	132.6	188.9	11	27	19
Triclosan	222.4	140.6	202.1	18	11	16
Zidovudine	226.9	103	172.5	10	25	16
